# NARROWING RACIAL AND ETHNIC DISPARITIES IN HOME AND COMMUNITY-BASED SERVICES

**DOI:** 10.1093/geroni/igac059.594

**Published:** 2022-12-20

**Authors:** Edem Hado

**Affiliations:** AARP, Washington, District of Columbia, United States

## Abstract

Older adults overwhelmingly prefer to age at home and in their community, yet research shows uneven access to quality home and community-based services (HCBS), especially among diverse racial and ethnic groups. Our research in Georgia and New York employs participatory research methods, including key informant interviews and focus groups conducted in 2021 and 2022, to identify root causes of disparities in access to, quality of, and experience with HCBS for older racial and ethnic and LGBTQI+ communities. Some root causes identified include: complexity in and lack of funding, tension between equal versus equitable service provision, and meaningful community outreach, particularly in Latino communities. We then identify scalable opportunities policy and programmatic interventions to improve care and service equity for older adults in those communities.

